# Cardiopulmonary Collapse during Labour

**DOI:** 10.1155/2010/707619

**Published:** 2010-08-01

**Authors:** Vasilis Sitras, Lasse Raatiniemi, Kristina Larsby, Claus Klingenberg

**Affiliations:** ^1^Women's Health and Perinatology Research Group, Institute of Clinical Medicine, Faculty of Health Sciences, University of Tromsø, 9037 Tromsø, Norway; ^2^Department of Obstetrics and Gynecology, Akershus University Hospital, Sykehusveien 25, 1478 Lørenskog, Norway; ^3^Department of Anesthesiology and Intensive Care, University Hospital of North Norway, 9038 Tromsø, Norway; ^4^Department of Cardiology, University Hospital of North Norway, 9038 Tromsø, Norway; ^5^Department of Pediatrics, University Hospital of North Norway and Institute of Clinical Medicine, University of Tromsø, 9037 Tromsø, Norway

## Abstract

Cardiopulmonary collapse during labour is a catastrophic event caused by various medical, surgical and obstetrical conditions. It is an emergency that threatens the life of the mother and her unborn child. We present a case of a pregnant woman who suffered from preeclampsia and underwent induction of labour. Severe lung edema occurred early in labour that caused cardiopulmonary collapse. Advanced heart-lung resuscitation was established immediately and continued until an emergency cesarean section was performed few minutes later. The outcome was favourable for both mother and child. We further discuss some aspects of the pathophysiology and appropriate treatment of cardiorespiratory arrest during labour, which involves the coordinated action of the obstetric, pediatric and surgical ward personnel.

## 1. Introduction

Cardiopulmonary collapse in labouring women is a rare condition that necessitates immediate and coordinated action of gynecologists, anaesthesiologists, and neonatologists in order to minimize maternal and neonatal morbidity and mortality. We present a case in which pulmonary edema led to cardio-respiratory arrest, where emergency cesarean section under cardiopulmonary resuscitation saved the life of both the mother and the infant.

## 2. Case Presentation

A 21-year-old, nulliparous woman was admitted at the Department of Obstetrics and Gynecology, University Hospital of Northern Norway, at 40 weeks' gestation because of preeclampsia. She had epilepsy in her medical history, but antiepileptic treatment was stopped in the second week of pregnancy. At the first antenatal visit her blood pressure (BP) was 106/67 mmHg. On admission her BP was 131/91 and 153/96 mmHg, measured 6 hours apart. She had 1+ protein on urinary dipstick and generalized edema; all blood tests were normal. She gained approximately 34 kg weight during pregnancy, and on admission her body mass index was 34.9. Two days before admission, she had experienced moderate attacks of dyspnea. Abdominal ultrasound for fetal assessment showed normal biometry, amniotic fluid index, and blood flow in the umbilical artery. Nonstress test was normal. 

Labour was induced with misoprostol until the cervix was favorable for rupture of membranes, two days later. Lumbar epidural analgesia, consisting of ropivacaine and fentanyl, was given uneventfully early in labour (2 cm cervical dilatation). An anaesthesiologist was consulted five hours later because of inadequate analgesia. Her BP was 160/102 mmHg. Fine crackles were present on chest auscultation. Respiratory rate was difficult to assess due to labour pains. Within the next twenty minutes, the patient became increasingly dyspnoic and agitated. Oxygen saturation was low (69%–82%) despite oxygen supplement given by mask. The next few minutes, the patient lost consciousness and became cyanotic with secretion of pink frothy sputum. Emergency cesarean section was decided. Cardiopulmonary resuscitation was started in the delivery room and was continued during transportation to the operation theatre. The patient was intubated without general anesthesia because of cardiovascular instability and reduced consciousness. When the endotracheal tube was inserted, anesthesia was induced with ketamine. Cesarean section was performed under nonsterile conditions on the patient's bed using a subumbilical midline incision. Anesthesia was continued by sevoflurane and fentanyl. Norepinephrine infusion was given to sustain circulation (mean BP of 65/42 mmHg), and high positive end-expiratory pressure and diuretics were used to relief pulmonary edema. Arterial blood gas analysis showed acidosis with: pH 7.13, PaO2 17.1 (Fio2 100), PaCO2 7.51, base excess (BE) –10.2, and Hb 7.8 g/L. 

An echocardiogram performed on arrival at the intensive care unit (ICU) showed findings of subnormal ejection fraction of the left ventricle (45%) as well as moderately reduced function of the right ventricle. Both ventricles had normal dimensions; there were no signs of elevated pressure on either side nor signs of valve pathology or pulmonary embolism. Ventilation was kept with pressure-supported respirator mode. A chest X-ray showed pleural effusion. Laboratory findings showed the following: ALAT 103 IU/L, ASAT 135 IU/L, Hct 27%, Hb 8.2 g/L, albumin 25.6 gr/L, and platelet count 248 000/L. The patient was extubated 15 hours after admission to the ICU. A control echocardiogram was performed two days and one year after delivery with normal findings.

A male baby was delivered 9 min after the cesarean section was decided. His birth weight was 3840 g, and Apgar scores were 0, 4, and 5 after 1,5 and 10 min, respectively. The umbilical artery blood gas analysis showed metabolic acidosis with pH 6.99, BE −12 mmol/L, and lactate 7.1 mmol/L. The baby was pale, floppy, not breathing, and had no obvious cardiac activity. He was immediately resuscitated and intubated. After 4 minutes, the heart rate was above 100/minute. However, during the first hours of life, he was neurologically abnormal with low tone. He therefore fulfilled our clinical inclusion criteria for therapeutic hypothermia which was performed for 72 hours without any complications. He was extubated after 2 hours and was thereafter breathing spontaneously. There were no clinical or electric seizures. MRI of the brain on day 4 was normal. Eleven days after delivery, both mother and child were discharged in good clinical conditions and good prognosis.

## 3. Discussion

There are several medical, and obstetrical conditions that can lead to cardiopulmonary collapse during labour. We believe that our patient had mild preeclampsia [1] as a predisposing factor leading to pulmonary edema that resulted in cardiopulmonary arrest. Other differential diagnoses have been considered such as amniotic fluid embolism which is a common cause of peripartum maternal death [2]. However, in our case, amniotic fluid embolism is unlikely to be the reason of cardiopulmonary collapse, because there were not serological coagulation disturbances or echocardiographic signs of pulmonary embolism [3]. Gestational hypertension might also be a possible differential diagnosis, however, our patient had normal blood pressure at first antenatal control and developed proteinuria during the late third trimester. Moreover, the patient's clinical condition got better quite soon after delivery, which also supports the diagnosis of preeclampsia. 

Pulmonary edema occurs in 2%–5% of women with preeclampsia [4], and these cases are classified as severe preeclampsia [5]. Pulmonary edema in pregnancy is often associated with medical (hypertension, diabetes), surgical (cesarean section, bleeding) and obstetric (preeclampsia, abruption placenta, CID) complications [6]. Pregnancy itself implies some physiologic changes such as anemia and increased intravascular volume. In addition, during preeclampsia, the increased vascular resistance and renal impairment predispose to further cardiopulmonary complications. Cardiopulmonary resuscitation in pregnant women is challenging due to the physiological anatomical changes, that is, supine heart position and aorto-caval compression of the pregnant uterus that reduces cardiac output and venous return. Moreover, tracheal intubation might be difficult because of laryngeal edema and increased risk of pulmonary aspiration [7]. 

Emergency caesarean section under cardiopulmonary resuscitation within 4 minutes of maternal cardiac arrest is the recommended management [8]. Perimortem cesarean is shown to not only improve neonatal prognosis, but also to release maternal vena caval compression and increase maternal cardiac output [9]. Thus, an alarm system for emergency cesarean section that implies coordinated action of a team of obstetricians, anesthesiologists, and neonatologists, is necessary to minimize time to delivery aiming for improved maternal and neonatal outcomes.
